# Growth, performance, and carcass characteristics of feedlot Holstein steers fed ractopamine hydrochloride^1^

**DOI:** 10.1093/tas/txz157

**Published:** 2019-10-04

**Authors:** Cathy L Lockard, Chris J Richards, Caleb G Lockard, Maggie Youngers, Mariah A Woolsoncroft, Taylor C Husz, Blake K Wilson, Carla L Goad, Todd A Jackson, Douglas L Step, Bryan C Bernhard, Marilyn J Corbin, Clint R Krehbiel

**Affiliations:** 1 Department of Animal and Food Science, Oklahoma State University, Stillwater, OK; 2 Department of Statistics, Oklahoma State University, Stillwater, OK; 3 Department of Veterinary Pathobiology, Oklahoma State University, Stillwater, OK; 4 Department of Veterinary Clinical Science, Oklahoma State University, Stillwater, OK; 5 Zoetis, Parsippany, NJ

**Keywords:** beta-adrenergic agonist, carcass quality, feedlot performance, Holstein steers, mobility, ractopamine hydrochloride

## Abstract

Growth-promoting technologies such as implants, ionophores, and β-agonists improve feedlot performance, efficiency, and carcass characteristics of cattle. The objective of this experiment was to determine the effects of dose and duration of ractopamine hydrochloride (RH) on feedlot performance and carcass characteristics when fed to Holstein steers. A randomized complete block design was used with a 3 × 3 factorial arrangement of treatments with 3 RH doses (0, 300, or 400 mg∙steer^−1^∙d^−1^) fed for 3 durations (28, 35, or 42 d). Holstein steers (*n* = 855; initial body weight [BW] = 448 ± 37 kg) were blocked by BW and randomly allocated to 1 of 9 pens (15 blocks; 9 dose × duration treatment combinations) approximately 72 d before harvest. Weekly pen weights, chute temperament scores, and animal mobility were determined during the RH feeding period. At harvest, carcass data were collected on all steers, and tenderness was measured on steaks from 3 or 4 randomly selected steers from each pen and slice shear force (SSF) was determined on one steak selected from each side of the carcass after aging for 14 or 21 d. For feedlot performance, carcass characteristics, and SSF, no dose × duration interactions were observed (*P* ≥ 0.11). With increasing RH dose, average daily gain (ADG) and gain-to-feed ratio (G:F) increased linearly (*P* ≤ 0.01), whereas BW gain increased linearly with RH dose and duration (*P* ≤ 0.01). Hot carcass weight (*P* = 0.02) and longissimus muscle (LM) area (*P* ≤ 0.01) increased linearly with increasing RH dose. The percentage of carcasses in the USDA Yield Grade 2 category increased linearly (*P* ≤ 0.01) and percentage of carcasses in the USDA Yield Grade 4 category tended (*P* = 0.08) to decrease linearly as RH dose increased. In the 14-d aged steaks, the percentage of steaks with SSF ≤ 15.3 kg decreased linearly (*P* ≤ 0.01), whereas the percentage of steaks with ≥20.0 kg SSF increased linearly (*P* ≤ 0.01) with increasing RH dose. After 21-d aging, there was a tendency (*P* = 0.06) for a greater percentage of steaks from steers fed RH to have SSF ≥ 20.0 kg (2% of total steaks), but no difference (*P* ≥ 0.12) in the percentage of steaks with SSF ≤ 19.9 kg. Final chute temperament (*P* ≥ 0.45) and animal mobility (*P* ≥ 0.67) scores were not affected by feeding RH. Increasing the dose of RH (300 or 400 mg∙steer^−1^∙d^−1^) fed for 28 to 42 d before harvest increased ADG, G:F, hot carcass weight, and LM area when fed to Holstein steers with no negative effects on behavior or mobility. The percentage of steaks classified as not tender improved when steaks were aged for 21 d from steers treated with RH.

## INTRODUCTION

Beta-adrenergic agonists (βAA) have been shown to improve feedlot performance and carcass characteristics of beef cattle when fed for 28 to 42 d before harvest. Ractopamine hydrochloride (RH; Actogain, Zoetis Parsippany, NJ) is a βAA that increases carcass leanness by increasing protein accretion and decreasing fat accretion (National Academies of Sciences, Engineering, and Medicine [NASEM], 2016). Ractopamine is to be fed at a rate of 90 to 430 mg·animal^−1^·d^−1^ for the final 28 to 42 d of the feeding period to improve rate of weight gain, feed efficiency, and carcass leanness.

According to the National Beef Quality Audit (Moore et al., 2012; Boykin et al., 2017), the percentage of Holstein steers in the fed cattle market increased from 10% to 16% from 2011 to 2016. Although demand varies depending on beef cow numbers, Holstein bull calves continue to be an integral part of the U.S. beef industry. Although previous research on the dosage and duration of RH in Holstein steers is limited compared with beef cattle, the addition of growth-promoting technologies such as implants and βAA provides a means to enhance meat production without decreasing meat quality.

Previous research has shown the addition of RH to the diet of beef or Holstein steers prior to harvest resulted in improvement in average daily gain (ADG), gain-to-feed ratio (G:F), hot carcass weight (HCW), and carcass leanness (Arp et al., 2014; Brown et al., 2014; Bittner et al., 2017). However, research determining the effects of feeding RH at varying doses and durations in Holstein steers is limited. The objective of this experiment was to characterize feedlot performance, carcass characteristics, behavior and mobility, and meat tenderness of Holstein steers fed RH at 0, 300, or 400 mg·steer^−1^·d^−1^ for 28, 35, or 42 d.

## MATERIALS AND METHODS

All animal care and management procedures for this experiment were approved by the Oklahoma State University Institutional Animal Care and Use Committee.

### Cattle

Holstein steers were obtained in 2 separate groups from a commercial feedlot located near Happy, TX, and transported 568 km to the Willard Sparks Beef Research Center (WSBRC) near Stillwater, OK. Group 1 arrived on 6 February 2015 (441 steers; initial body weight [BW] = 454 ± 37 kg), and Group 2 arrived on 9 September 2015 (414 steers; initial BW = 438 ± 35 kg). Steers were used in a randomized complete block experimental design with a 3 × 3 factorial arrangement of treatments where RH was fed at 3 doses for 3 durations. Steers were blocked by BW and randomly allocated within block to 1 of 9 pens. Each pen within each block was then randomly assigned to 1 of 9 treatments. Ractopamine hydrochloride was fed at 0 (CON), 300, or 400 mg·steer^−1^·d^−1^ for 28, 35, or 42 d before harvest. Group 1 had 7 blocks (63 steers per block; 21 steers per dosage; 21 steers per duration per block; and 7 steers per pen). Group 2 had 8 blocks (45 to 54 steers per block; 15 to 18 steers per dosage per block; 15 to 18 steers per duration per block; and 5 or 6 steers per pen).

### Arrival Processing

Upon arrival, steers were collectively weighed on a pen scale to obtain an average BW and placed into 16 holding pens (25 to 27 steers per pen) that were 12 × 30 m. Pens were soil surfaced with 12 m of concrete fence-line bunk and a 75-L concrete fence-line water tank shared between 2 adjacent pens. Approximately 36 h following arrival, steers were individually moved through a squeeze chute where BW and hip height were measured and steers were identified with both visual and electronic identification ear tags. Steers were vaccinated with a modified-live viral vaccine (Bovi Shield IBR; Zoetis, Parsippany, NJ) to prevent bovine respiratory disease caused by infectious bovine rhinotracheitis and treated for internal and external parasites with fenbendazole oral anthelmintic (Safeguard; Merck Animal Health, DeSoto, KS) and a pour-on anthelmintic (Dectomax Pour-on; Zoetis). Steers were sorted based on the group’s individual median BW into heavy and light pens and were returned to their holding pens (25 to 27 steers per pen) after processing.

### Arrival Groups

On day 29 from arrival, steers within Group 1 and Group 2 were weighed individually, implanted with 40 mg of estradiol and 200 mg of trenbolone acetate (Revalor-XS, Merck Animal Health) and returned to holding pens. Due to a malfunction with a load cell on the chute scale, BW were not recorded for steers in Group 2. On day 31, all steers in Group 2 were weighed and individually and returned to holding pens. Steers were projected to a weight block from the day 29 or 31 BW, and final harvest dates were projected. Weight blocks were randomly assigned to 9 continuous pens located on either the south or north side of the feedlot. There were 64 treatment pens (32 pens per north and 32 pens per south). Block was randomly assigned to 9 pens located on either the north or south side of the feedlot. For the entire experiment, there were 6 blocks housed on the south and 9 blocks housed on the north. Steers were randomly allocated to 1 of the 9 treatment pens within a block and pens within a block/location were randomly assigned to 1 of 9 treatments. Upon allocation to their experimental pens, all steers received a colored ear tag unique to RH dosage. On d 30, all steers in Group 1 were sorted into their experimental pens. For Group 2, steers within a similar weight block were weighed individually 73 d prior to their projected harvest date. On the following d (d 72), all steers were sorted to their experimental pens.

Finishing pens were 4.5 × 15 m in area with a 4.5-m-long concrete bunk at the front of the pen. The pens contained a 4.5 × 4.5 m concrete pad, with the remainder of the pen being soil surfaced. The pens were under partial cover, with the bunk and pad being covered by an overhang. A 75-L concrete water tank (model J 360-F; Johnson Concrete, Hastings, NE) was shared between 2 pens and was cleaned 3 times/wk.

The experiment was designed with a minimum 28-d treatment pen adaptation prior to the start of RH. During the beginning of Group 1’s experiment period, the feedlot received a high amount of rainfall. Due to deteriorating pen conditions, steers that had not begun their RH treatment period (the lightest 4 blocks) were moved to larger holding pens for approximately 21 d. Steers were penned by RH dose within block. A minimum of 14 d before the start of the RH treatment period for the 42-d duration steers, steers in each block were weighed, sorted, and returned to their original treatment pens.

### Health Management

Steers were observed daily in the pens by a trained evaluator blinded to treatments. Steers were evaluated based on a modified DART (depression, appetite, respiration, and temperature) system (Zoetis, Parsippany, NJ) with some modifications as described by Step et al. (2008). Signs of depression included, but were not limited to depressed attitude, lowered head, glazed or sunken eyes, slow or restricted movement, arched back, difficulty standing or walking, knuckling of joints or dragging toes when walking, or stumbling. Signs of abnormal appetite included an animal that was completely off feed, an animal eating less than expected or eating slowly, a lack of gut fill or gaunt appearance, and/or obvious BW loss. Respiratory signs included labored breathing, extended head and neck (in an attempt to breathe), and/or audible noise when breathing. Steers were evaluated based on the 0 to 4 severity scoring system adapted from Step et al. (2008) and Wilson et al. (2016). The evaluators assigned a steer a severity score from 0 to 4 based on clinical signs and severity of those observed signs. A score of 0 was assigned for a calf that appeared clinically normal. A score of 1 was assigned for mild clinical signs, 2 for moderate clinical signs, 3 for severe clinical signs, and 4 for a moribund animal. For a steer to be assigned a score of 4, the steer had to be unable to rise, had to require assistance to rise, or had to have extreme difficulty standing, walking, or breathing. Steers with severity score of 4 required immediate attention. All steers assigned a severity score 1 to 4 were taken to the processing chute for rectal temperature measurement (GLM-500; GLA Agricultural Electronics, San Luis Obispo, CA), unless it was deemed necessary for moribund steer to receive treatment in the home pen. Any animal that was identified with a severity score of 1 or 2 and had a rectal temperature of 40°C or greater received an antimicrobial according to label instructions. All antimicrobials were administered subcutaneously per manufacturer’s label directions following Beef Quality Assurance Guidelines (National Cattlemen’s Beef Association [NCBA], 2001).

Steers pulled for other health reasons (e.g., lameness issues or pink-eye) were moved to the squeeze chute and evaluated by a trained individual. Steers were evaluated for lameness based on a 1 to 4 scale: 1 = slight lame; 2 = mildly lame; 3 = moderately lame; 4 = severely lame (Step et al., 2008). If the veterinarian deemed necessary, steers were treated subcutaneously with oxytetracycline (4.5 mL/45.4 kg BW; Bio-Mycin; Boehringer Ingelheim, St. Joseph, MO) and intravenously with flunixin meglumine (1.0 to 1.5 mL/45.4 kg BW; Banamine; Merck Animal Health, DeSoto, KS).

If steers were unable to continue due to health reasons before or during the experimental period, they were removed from their home pen, evaluated by a veterinarian, and euthanized for humane reasons, if deemed necessary. All steers that died or were euthanized were taken to the Oklahoma Disease Diagnostic Laboratory at the Center for Veterinary Health Sciences for a complete necropsy.

### Feed and Bunk Management

Receiving and finishing diets were formulated to meet or exceed the National Research Council (NRC, 2000) requirements (Table 1). Upon arrival, steers received 0.50 kg per steer of prairie hay and 3.2 kg per steer of the receiving diet. The following day, steers received 3.2 kg per steer of the receiving diet and 3.2 kg per steer of the finishing diet. Steers were transitioned to the finishing diet over a 7-d period by decreasing the receiving diet by 0.5 kg each day and adjusting the total feed delivered by increasing the finishing diet. Steers were fed twice daily at approximately 0700 and 1300 h. Feed bunks were managed to ensure trace amounts of feed were in the bunk before morning feeding. Each morning, bunks were cleaned to remove in-edible feed, manure, etc. Bunk dividers were installed in an attempt to ensure no cross-contamination or cross-feeding occurred. Feed was mixed and delivered in a 274-12 Roto-Mix mixer wagon (Roto-Mix; Dodge City, KS) with delivery accuracy to the nearest 0.50 kg. Feed refusal were removed from the feed bunk and weighed on weigh days, or if feed was wet, or if feed was more than 1-d old.

**Table 1.** Finishing diet ingredients, supplement, and composition fed to Holstein steers with ractopamine hydrochloride^1^

Item		Receiving^2^	Finishing
Ingredient, %	Steam flaked corn^3^	58.49	61.60
	Dried distillers grain plus solubles	16.49	16.47
	Alfalfa hay	5.77	4.27
	Prairie hay	10.20	8.65
	Feed fat	3.45	3.45
	Water	0.10	0.11
	Dry supplement^4^	5.50	5.50
Composition^5^			
	NE_m_, Mcal/kg		0.95 ± 0.04
	NE_g_, Mcal/kg		0.65 ± 0.03
	CP, %		13.35 ± 0.66
	ADF, %		11.53 ± 1.23
	NDF, %		21.73 ± 2.29
	Fat, %		7.22 ± 0.45
	Calcium, %		0.67 ± 0.12
	Phosphorus, %		0.37 ± 0.03
	Magnesium, %		0.18 ± 0.01
	Potassium, %		0.66 ± 0.04

^1^Ractopamine hydrochloride (RH) was fed at a rate of 0, 300, or 400 mg·steer^−1^·d^−1^ for 28, 35, or 42 d at the end of the feeding period. The RH was mixed with 0.5 kg of dried distillers grain plus solubles (DDGS)/steer and top dressed with the morning feeding. The amount of RH/DDGS mix replaced the appropriate amount of DDGS in the diet.

^2^Recieving diet was fed when steers arrived at feedlot and for a consecutive 7 d during transition onto the finishing diet. Finishing diet was fed for an average of 150 d.

^3^Starch availability, 50%; total starch, 74.35%; flake weight, 11 kg/bushel.

^4^Supplement ingredients: ground corn, 36.10%; limestone, 28.50%; wheat midds, 19.75%; urea, 6.50%, magnesium oxide, 0.96%; zinc sulfate, 0.58%; salt, 0.36%; copper sulfate, 0.11%; manganese oxide, 0.11%; selenium pre-mix, 0.05%; vitamin A (30,000 IU/g), 0.29%; vitamin E (500 IU/g), 0.08%; monensin, 0.45%; tylosin, 0.23%. Monensin and tylosin (Rumensin and Tylan, respectively, Elanco Animal Health) were fed at a calculated rate of 48.8 and 9.5 mg/kg daily, respectively.

^5^NE_m_ = net energy for maintenance; NE_g_ = net energy for gain; CP = crude protein; ADF = acid detergent fiber; NDF = neutral detergent fiber.

The finishing ration contained steam-flaked corn, dried distiller’s grains plus solubles (DDGS), alfalfa and prairie hay, liquid feed fat, and dry supplement (Table 1). The dry supplement was pelleted and contained ground corn, wheat midds, minerals, vitamins, monensin sodium (48.8 mg/kg of feed), and tylosin phosphate (9.5 mg/kg of feed; Rumensin and Tylan, respectively, Elanco Animal Health, Greenfield, IN).

Group 1 diet samples were collected on the Wednesday of each week from all pens of steers housed in the north and south barns and composited by each RH dose treatment. For Group 2, diet samples were collected on Wednesday of each week from all pens of steers housed in the north and south barns. For Group 2, diet samples were composited separately for each block and RH dose treatment. Diet samples were composited for each block by RH dose and analyzed at a commercial laboratory (Servi-Tech, Dodge City, KS). Means and SD for diet composition are reported in Table 1.

Samples were dried in a forced-air oven for approximately 72 h at 60°C to determine dry matter (DM). Dry matter intake (DMI) for each pen was calculated by dividing total kg of feed delivered by total head days for each pen, and weekly DM were used to calculate average daily DMI. After refusals were collected from the bunk, a subsample was placed in the same forced-air over for approximately 72 h to calculate DM. Refusals were subtracted from the weekly feed on a DM basis.

### Experimental Period

The RH was mixed with 0.5 kg DDGS per steer and top dressed with the morning feeding. The appropriate amount of RH for each treatment pen was weighed daily by 1 of 2 trained individuals. Ractopamine hydrochloride was weighed on a gram scale with ±0.02 g accuracy and mixed with DDGS for 5 min in a cement mixer (Kushlan Concrete Mixer; Sugar Land, TX). The gram scale used for daily RH was validated to 75 g (±0.02 g) on each Wednesday throughout the RH feeding period. Three cement mixers were used, with each mixer designated for CON, 300, or 400 mg RH treatments. At randomization to treatment pen, each pen was assigned a unique color for dose and a unique color for duration. Each treatment pen had an individually labeled bucket with block number and color combination for dose and duration to prevent errors while top-dressing. Immediately following each pen’s morning feed delivery, the DDGS/RH mixture was top dressed by 2 or 3 trained individuals. Steers were not allowed access to the bunk until the top dress was fully mixed with the diet. The total diet fed to calves allowed for the additional 0.5-kg inclusion of DDGS by decreasing the DDGS in the ration by 0.50 kg per steer.

On day 0 of the respective treatment duration, individual and pen weights were recorded. Thereafter, pen weights were recorded every 7 d until harvest. The individual chute scale was validated within 1.8 kg in the morning before all processing weigh days and the pen scale was certified by the State of Oklahoma before steers arrived to the feedlot. Steers on the first 5 blocks had individual and pen weights measured on day 42 and then were loaded on trucks for shipment. The final 10 blocks had individual weights measured on day 40, and pen weights were recorded before loading trucks for shipping on day 42. This day 42 or day 40 individual BW was used as the final BW for all blocks. Each block was split between 2 trucks with the first 4 ½ pens on truck 1 and the last 4 ½ pens on truck 2. Pens of steers were loaded on trucks following the same order as randomization of pen to treatment to prevent treatment bias associated with trucking. Once loaded, steers traveled approximately 435 km to the abattoir (Cargill Meat Solutions; Dodge City, KS), and cattle from both trucks were unloaded into a cement-based holding pen for approximately 3 to 4 h before harvesting.

### Behavior

Chute temperament was recorded on all steers during allocation to treatment pens, on d 0 of their respective duration, and at the end of the RH feeding period. When steers entered the squeeze chute, heads were caught and they were restrained, while the observer recorded the chute temperament score. Steers were evaluated based on a 4-point scale: 1 = calm, no movement; 2 = restless, shifting; 3 = squirming, occasionally shaking the squeeze chute; and 4 = continuous vigorous movement and shaking of the squeeze chute (Grandin, 1995; Voisinet et al., 1997; Bernhard, 2014). After the 15-s chute temperament observation, steers were evaluated upon exiting the squeeze chute. Exit score was evaluated using a 4-point scale: 1 = walk; 2 = trot; 3 = run; and 4 = jump (Lanier and Grandin, 2003; Vetters et al., 2013; Bernhard, 2014).

### Mobility

Mobility was observed on days that steers were individually processed through the squeeze chute, on day 0 of the respective RH duration start date, and at the end of the RH feeding period. A video camera (Samsung HMX-F90; Samsung Town, Seoul, Korea) recorded the cattle at a 90° angle as they individually walked down the alleyway approximately 10 m from the chute. Distance between reference points was measured and marked with tape prior to processing the steers. From the video footage, stride length was measured using a freeze-frame of each steer by measuring the distance between the furthest back rear foot to the back of the forward rear foot when both hooves were in contact with the dirt surface. The freeze frames were analyzed and length was quantified using ImageJ software (http://imagej.nih.gov/ij/) to compare the distance between the 2 rear hooves to the distance between 2 known reference points. From the same videos, an individual mobility score was assigned. Mobility was scored based on a 4 point scale: 1 = normal, long, fluid strides, and weight bearing on all 4 feet; 2 = slightly hesitant and stiff, shuffles feet, but still moves with the herd; 3 = obviously stiff and sore footed, reluctant to move, cannot keep up with the herd; 4 = reluctant to move, refuses to move even when encouraged by a handler, steps are short and very unsteady (Lily Edwards-Callaway; JBS, Greeley, CO). Pen and feedlot conditions were muddy due to an abnormal quantity of rainfall during Group 1 making it difficult for cattle to move “normally” and for the evaluator to score the mobility of the steers. Therefore, only data from Group 2 were included in the mobility analyses.

On the morning of shipment, pen mobility was evaluated as steers were moved to the pen scale prior to loading onto trucks using the same 4 point scale described earlier. As steers were individually weighed, a colored spray-painted blotch (color unique for pen within block) was applied to the tail head and the base of the left ear of each steer. The unique colors were used for pen identification (treatment identification) at the harvest facility. Steers were shipped to Cargill Meat Solutions, Dodge City, KS. Steers were shipped at approximately 0700 h, unloaded at the harvest facility at approximately 1200 to 1300 h and stunned at approximately 1500 to 1600 h. Steers were moved from holding pens to the abattoir (approximately 76 m) by a trained handler. As steers were moved out of the holding pens, individual animal mobility was evaluated as previously described using the unique color spray paint to identify animals within treatment.

### Carcass Collection

Carcass data were collected on all steers through Cargill Meat Service (Dodge City, KS). Hot carcass weight, liver abscess scores, and condemned gastrointestinal tracts were recorded. Liver abscesses were scored as no abscesses (O), 1 to 4 small active abscesses (A), and 1 or more large, active abscesses (A^+^; Brink et al., 1990). Carcasses were chilled 40 to 48 h, ribbed at the 12th rib, and evaluated for marbling, yield grade, fat thickness, and longissimus muscle (LM) area. Marbling scores were used to assign a quality grade to all carcasses.

The fourth block of steers was loaded and shipped on 12 August 2015. Due to notification from the abattoir of a potential RH residue from the third block of steers shipped, block 4 steers were returned to the feedlot. Trucks moved approximately 30 min out of Stillwater, OK, and steers were on trucks for approximately 60 to 90 min before being returned to their original treatment pens. They were fed the same amount of DM as the prior day without RH through reshipment on 19 August 2015. The experiment investigator and personnel from WSBRC reviewed all feed records, ration/supplementation calculations, and mixing protocols, and were not able to identify an error in RH delivery. As a precaution, a 48-h RH withdrawal period prior to harvest was established for all subsequent blocks of steers enrolled in the experiment. Block 4 live performance data were included in live data analyses, but carcass data were removed from carcass data analyses due to the steers being shipped at a different time.

Three or 4 steers from each pen were randomly selected for slice shear force (SSF) tenderness sampling. Carcasses were sent to designated rails for further sample collection. One steak, approximately 3.8 cm thick was removed from the anterior edge of the short loin, 13th rib end, from both sides of the carcass. Steak samples were tagged individually, sealed, and shipped from the Dodge City packing facility to the Cargill Innovation Center (Dodge City, KS). One sample was aged for 14 d and the other for 21 d. After aging, both samples were frozen. Subsequently, all samples were cooked and tested on the same day to avoid day-to-day variation. Due to unforeseen timing errors within the packing facility, only 11 steak samples were collected for Block 1; therefore, Block 1 was excluded from analyses. Excluding Blocks 1 and 4, 432 carcasses were randomly selected to be sampled prior to shipping. Due to plant miscommunication, 15 carcasses, of all treatments, were missed and data for 417 carcasses were received for SSF tenderness analyses.

Immediately following aging, steaks were vacuumed packaged and stored at −28.9°C until steaks were thawed and sampled. Samples were thawed at room temperature for approximately 24 to 36 h, cut to 2.5 cm thick, and weighed for a raw weight before cooking. Before cooking, internal temperature of steaks was between 35 and 37°C. Samples were cooked in a Lincoln Impingement Oven (Fort Wayne, IN) at 71°C for 14 min and 30 s to ensure the 71°C end point was reached. Internal cook temperature was measured by a Calibrated Thermometer (Digi-Sense, Vernon Hills, IL). After cook testing, a slice of each sample was removed from the lateral end that was 1.0 cm thick, 5.1 cm long, and parallel to the muscle fibers. Samples were analyzed and tested for SSF using a Texture Analyzer TAXT2i (Brewster, NY). According to the American Society for Testing and Materials (ASTM, 2011) classifications, qualifying meat cuts with SSF values that are ≥20.0 kg are considered not tender or tough; SSF values between 15.4 and 19.9 are considered tender; and SSF values ≤ 15.3 kg are considered as very tender.

### Statistical Analysis

Data were tested for normality using PROC UNIVARIATE of SAS (SAS 9.4, SAS Inst. Inc., Cary, NC). All steer performance, carcass characteristics, and SSF data were analyzed with the MIXED procedure of SAS with pen serving as the experimental unit. Weight block was included as a random effect, and the model statement included dose, duration, and the dose × duration interaction. The USDA Quality Grade, USDA Yield Grade, liver abscess scores, behavior, and mobility data were analyzed using the PROC GLIMMIX procedure of SAS with pen as the experimental unit. To account for differences in day 0 behavior or mobility, day 0 scores were used as a covariate in the model. Block was used as a random effect, and the model statement included dose, duration, and the dose × duration interaction. For pen mobility data, week and its interactions with dose and duration were also included in the model. To test for trends where there were no interactions, linear and quadratic orthogonal contrasts were used for RH dosage and duration.

Least squares means were considered significantly different when *P* < 0.05 and a trend was declared when 0.05 ≥ *P* ≤ 0.10. When the *F*-test was *P* < 0.10 and no dose × duration interaction was observed, contrasts were used to test for linear and quadratic effect of dose and duration.

## RESULTS

During the RH feeding period, 8 steers died and another 8 steers were removed from the experiment due to health-related issues. Five steers from Group 1 died (1 bloat, 1 injury, and 3 respiratory disease), and 7 steers were removed due to lameness. Three steers from Group 2 died (2 bloats and 1 injury), and 1 steer was removed due to lameness. Eight-hundred and thirty-nine steers completed the experiment. Nine steers were removed from the CON, 5 steers from 300 mg, and 2 steers from 400 mg.

### Performance

There were no dose × duration interactions (*P* ≥ 0.29) for performance response variables measured in this experiment. Therefore, main effects least squares means are shown (Table 2). Dose of RH did not have an effect on d 0 BW (*P* ≥ 0.35), but by experimental design, there was a linear decrease (*P* ≤ 0.01) in d 0 BW with increasing duration of feeding. Final BW tended (linear effect, *P* = 0.07) to increase with increasing RH dose, but was not affected (*P* ≥ 0.87) by duration. There were linear dose (*P* = 0.003) and duration (*P* ≤ 0.01) effects on live BW gain during the RH feeding period. Steers gained 6.3 and 7.2 kg more when fed 300 or 400 mg·steer^−1^·d^−1^ of RH, respectively, than when CON was fed. As a result of differences in starting weight (by experimental design), steers on the 35-d duration gained an average of 7.3 kg more than the 28-d duration steers, and steers on the 42-d duration gained an average of 13.6 kg more than steers on the 28-d duration.

**Table 2.** Effects of feeding ractopamine hydrochloride on body weight (BW)^1^ and performance of Holstein steers

	Dose				*P*-value		Duration				*P*-value	
Item	0	300	400	SEM	Linear	Quadratic	28	35	42	SEM	Linear	Quadratic
Pens	15	15	15				15	15	15			
Total steers	276	280	283				280	281	278			
BW, kg												
Arrival^2^	423	420	422	8	0.36	0.26	420	423	422	8	0.56	0.32
Day 0	599	598	596	5	0.35	0.69	605	598	591	5	<0.001	0.90
Final^3^	635	641	640	6	0.07	0.43	639	639	639	6	0.87	0.97
BW gain, kg^4^	36.4	42.7	43.6	3.8	0.003	0.51	34.1	41.4	47.7	3.8	<0.001	0.97
ADG, kg/d^5^	1.03	1.23	1.25	0.11	0.002	0.58	1.21	1.17	1.14	0.11	0.32	0.90
DMI, kg/d^6^	9.50	9.52	9.58	0.23	0.73	0.82	9.72	9.56	9.36	0.23	0.05	0.91
G:F^7^	0.106	0.128	0.129	0.010	0.002	0.64	0.123	0.121	0.121	0.010	0.75	0.82
Carcass-adjusted performance^8^												
Final BW, kg	635	640	641	7	0.04	0.89	639	639	638	7	0.68	0.83
BW gain, kg	35.6	42.4	44.3	3.7	0.002	0.91	33.9	41.3	47.1	3.7	<0.001	0.75
ADG, kg	1.02	1.21	1.28	0.11	0.001	0.99	1.21	1.18	1.12	0.11	0.29	0.84
G:F	0.106	0.127	0.132	0.012	0.004	0.86	0.123	0.123	0.120	0.012	0.73	0.96

^1^A calculated shrink of 4% was applied to all BW measurements.

^2^Arrival BW taken approximately 36 h after arrival to the feedlot.

^3^End of ractopamine feeding period after a duration of 28, 35, or 42 d.

^4^BW = body weight. BW gain calculated as follows: (final BW − day 0 BW).

^5^ADG = average daily gain. ADG from start to end of ractopamine hydrochloride feeding period.

^6^DMI = dry matter intake. DMI from start to end of ractopamine hydrochloride feeding period.

^7^G:F = gain-to-feed ratio. G:F calculated as follows: (ADG/DMI).

^8^Calculated from live weight adjusted for a 60.41% common dressing percentage. Data included all 15 blocks.

Feeding 300 and 400 mg∙hd^−1^∙d^−1^ RH, respectively, increased (linear effect, *P* ≤ 0.01) ADG by 0.20 and 0.22 kg/d, increased (linear effect, *P* ≤ 0.01) G:F by 20.8% and 21.7%, but did affect (*P* ≥ 0.73) DMI (Table 2). Duration of RH feeding did not affect ADG or G:F (*P* ≥ 0.32), but DMI was decreased (linear effect, *P* = 0.05) with increasing duration. Similar to live performance, increasing RH dose linearly increased carcass-adjusted final BW (*P* = 0.04), kg of BW gain (*P* ≤ 0.01), daily gains (*P* ≤ 0.01), and G:F (*P* ≤ 0.01). Duration of RH feeding did not affect carcass-adjusted ADG or G:F (*P* ≥ 0.29), but carcass-adjusted BW gain increased (linear effect, *P* ≤ 0.01) as duration of feeding increased, which is a result of experiment design.

### Carcass Characteristics

There were no dose × duration interactions (*P* ≥ 0.11) for carcass characteristics. Hot carcass weight increased linearly (*P* = 0.02) with increasing dose of RH but was not affected by duration of feeding (*P* = 0.57; Table 3). Dressing percent (*P* ≥ 0.31) and 12th-rib fat thickness (*P* ≥ 0.17) were not affected by dose or duration of RH feeding. Longissimus muscle area increased (linear effect, *P* = 0.001) as RH dose increased, but there was no effect of duration (*P* = 0.26) on LM area. The ratio of LM area to HCW was not affected by duration (*P* ≥ 0.34) but increased (*P* ≤ 0.01) as dose of RH increased. Marbling score decreased (linear effect, *P* = 0.03) as RH dose increased. In addition, marbling score was greater (quadratic effect; *P* = 0.04) for the 35-d duration steers than the 28- or 42-d duration steers. There were no effects of RH dose (*P* ≥ 0.16) or duration of feeding (*P* ≥ 0.19) on USDA Quality Grades. Calculated yield grade decreased linearly (*P* = 0.003) as dose of RH increased. In addition, percentage of steers in the USDA Yield Grade 2 category increased linearly (*P* ≤ 0.01), and percentage of steers in the USDA Yield Grade 4 category tended (linear effect, *P* = 0.08) to decrease as dose of RH increased. Duration of RH feeding did not affect (*P* ≥ 0.28) USDA Yield Grade.

**Table 3.** Effects of feeding ractopamine hydrochloride on carcass characteristics in Holstein steers

	Dose			*P*-value			Duration				*P*-value	
Item	0	300	400	SEM	Linear	Quadratic	28	35	42	SEM	Linear	Quadratic
Pens	14	14	14				14	14	14			
Total steers	256	260	263				260	261	258			
HCW^1^, kg	383	386	387	4	0.02	0.81	385	386	384	4	0.57	0.57
Dressing percent, %	60.2	60.3	60.4	0.5	0.31	0.64	60.3	60.3	60.3	0.5	0.91	0.63
Marbling scores	484	471	466	8	0.03	0.65	470	483	468	8	0.70	0.04
LM area^2^, cm^2^	78.0	79.3	80.7	0.7	0.001	0.53	80.0	78.7	79.4	0.7	0.26	0.44
LM/HCW, cm^2^/kg	0.204	0.207	0.209	0.002	0.001	0.73	0.208	0.205	0.206	0.002	0.46	0.34
12th rib fat thickness, cm	0.88	0.86	0.84	0.02	0.17	0.98	0.85	0.87	0.86	0.02	0.58	0.43
Calculated yield grade	3.21	3.12	3.07	0.06	0.003	0.72	3.09	3.17	3.14	0.06	0.34	0.37
USDA Quality Grade^3^												
Prime, %	0.8	0.0	0.4	0.1	0.28	0.30	0.0	0.8	0.4	0.1	0.45	0.19
Choice, %												
High, %	12.5	8.8	9.0	2.3	0.22	0.72	9.8	12.5	8.0	2.3	0.58	0.19
Low, %	70.7	66.9	69.3	3.0	0.59	0.47	69.3	67.4	70.2	3.0	0.84	0.53
Select, %	16.0	24.3	21.3	3.0	0.16	0.18	21.0	19.3	21.5	3.0	0.88	0.54
USDA Yield Grade												
YG 1, %	3.5	2.9	3.4	1.4	0.86	0.74	3.9	3.3	2.5	1.4	0.35	0.98
YG 2, %	32.3	40.9	42.6	4.1	0.008	0.82	42.0	36.1	37.6	4.1	0.28	0.30
YG 3, %	56.8	52.5	50.2	3.6	0.11	0.86	50.7	54.3	54.5	3.6	0.36	0.64
YG 4, %	7.5	3.8	4.0	1.9	0.08	0.61	3.4	6.4	5.5	1.9	0.36	0.32
Liver abscess^4^												
A, %	24.4	17.8	21.1	2.8	0.19	0.21	22.8	17.2	23.3	2.8	0.89	0.07
A^+^, %	36.4	36.7	39.2	4.0	0.58	0.63	37.1	37.8	37.4	4.0	0.95	0.89
O, %	37.8	43.3	37.9	4.4	0.68	0.19	37.6	43.5	37.8	4.4	0.97	0.14
Other, %	1.7	2.3	2.1	1.1	0.69	0.79	2.7	1.5	1.8	1.1	0.46	0.49

^1^HCW = hot carcass weight.

^2^LM = longissimus muscle.

^3^Quality grade assigned based on carcass marbling scores: <400—select; 401 to 600—low choice; 601 to 800—high choice; >801—prime.

^4^Liver abscess scoring was adapted from Brink et al. (1990). Livers were scored as follows: No abscesses (O), 1 to 4 small active abscesses (A), or one or more large, active abscesses (A^+^).

Calculated carcass gain and performance data are shown in Table 4. For calculated carcass performance, day 0 HCW was predicted as day 0 BW × 0.5975. Carcass gain increased (linear effect, *P* ≤ 0.01) as dose of RH and duration of feeding increased. In addition, carcass ADG (*P* ≤ 0.01) and carcass G:F (*P* ≤ 0.01) increased as dose of RH increased. As result of experimental design, calculated day 0 HCW (linear effect, *P* ≤ 0.01) and carcass ADG (linear tendency, *P* = 0.07) decreased as duration of feeding increased. Carcass G:F was not affected (*P* ≥ 0.30) by duration of feeding.

**Table 4.** Effects of feeding ractopamine hydrochloride on carcass gain in Holstein steers

	Dose				*P*-value		Duration				*P*-value	
Item	0	300	400	SEM	Linear	Quadratic	28	35	42	SEM	Linear	Quadratic
Actual HCW, kg	382.7	385.6	387.1	3.98	0.02	0.81	385.4	385.7	384.3	3.98	0.57	0.57
Initial HCW, kg^1^	356.9	356.2	355.6	3.25	0.40	0.71	360.9	356.0	351.9	3.25	<0.001	0.77
Carcass gain, kg^2^	25.5	29.6	31.3	2.57	0.001	0.34	24.3	29.6	32.4	2.57	<0.001	0.41
Gain over 0, kg	—	4.1	5.8				—	5.3	8.1			
Carcass ADG, kg/d^3^	0.73	0.85	0.91	0.07	0.001	0.31	0.87	0.85	0.77	0.07	0.07	0.56
Carcass G:F^4^	0.076	0.089	0.095	0.008	0.002	0.98	0.090	0.088	0.082	0.008	0.30	0.53

^1^Initial HCW calculated as follows: day 0 BW × 59.75%. HCW = hot carcass weight.

^2^Carcass gain calculated as follows: (actual HCW − predicted HCW).

^3^Carcass ADG calculated as follows: (carcass gain/duration). ADG = average daily gain.

^4^Carcass G:F calculated as follows: (carcass ADG/DMI). G:F = gain-to-feed ratio; DMI = dry matter intake.

### Tenderness-Slice Shear Force

**Figure 1.**
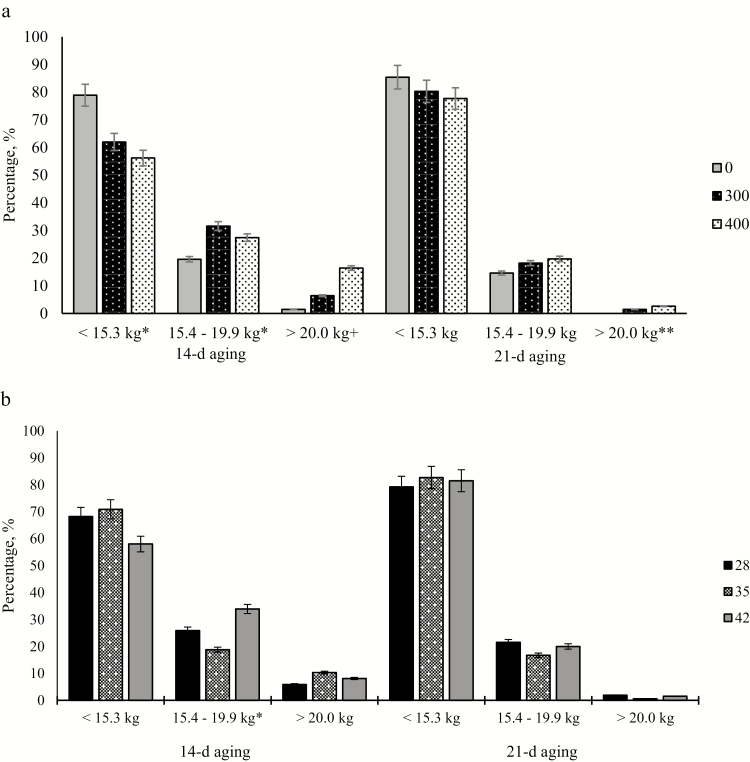
Effect of ractopamine hydrochloride dose (a) and duration (b) on slice shear force (SSF) distribution after 14- or 21-d aging. *Linear effect when *P* < 0.05. ^+^Quadratic effect when *P* < 0.05. **Linear tendency when 0.05 < *P* ≥ 0.10. Slice shear force was collected on 3 to 4 randomly selected steers from each pen (*n* = 417). Ractopamine hydrochloride was fed at 3 doses (0 [CON], 300, or 400 mg hd^−1^ d^−1^) at 3 durations (28, 35, or 42 d). Values separated by ASTM (2011) specifications; <15.3 kg (very tender); 15.4 to 19.99 (very tender to tender); >20.0 kg (tough; not tender). 14-d aging; <15.3 kg SEM = 4.89; 15.4 to 19.99 kg SEM = 4.21; >20.0 kg SEM = 2.81. 21-d aging; <15.3 kg SEM = 4.57; 15.4 to 19.9 kg SEM = 4.83; >20.0 kg SEM = 0.98.

At 14-d aging, there was a dose × duration interaction (*P* = 0.02) for the percentage of steaks with SSF values ≥ 20.0 kg. For RH dosages of 0, 300, and 400 mg, means were 0.0, 7.1, and 10.5 when fed for 28 d; 2.6, 1.9, and 26.5 when fed for 35 d; and 1.9, 10.3, and 12.2 when fed for 42 d, respectively. The 400 mg RH dose had the greatest percentage of steaks with SSF values equal to or above 20.0 kg. Steers fed 300 mg·steer^−1^·d^−1^ of RH had an intermediate percentage of steaks with SSF equal to or above 20.0 kg for the 28- and 42-d durations but had the lowest percentage of steaks with SSF equal to or above 20.0 kg for the 35-d duration. In addition, there was a dose × duration interaction (*P* = 0.05) for average SSF at 14-d aging (data not shown). Control steers had the least SSF values for all durations. Steers fed 400 mg·steer^−1^·d^−1^ of RH had the greatest average SSF values for the 35-d duration, whereas average SSF was greater for steers fed 300 mg·steer^−1^·d^−1^ for the 28- and 42-d durations. When steers were fed increasing RH doses, there was a linear (*P* ≤ 0.01) increase in steaks with SSF values ≥ 20.0 kg and a linear decrease (*P* ≤ 0.01) in the percentage of steaks with SSF ≤ 15.3 kg (Fig. 1a). As feeding duration increased, there was a tendency (*P* = 0.07) for a linear decrease in steaks with ≤15.3 kg SSF (Fig. 1b). In addition, there was a quadratic effect (*P* = 0.01) of duration for the 14-d aged steaks with an SSF between 15.4 and 19.9 kg. Steers fed for the 35-d duration had the least percentage of steaks with SSF between 15.4 and 19.9 kg than steers fed for 28 or 42 d.

**Figure 2.**
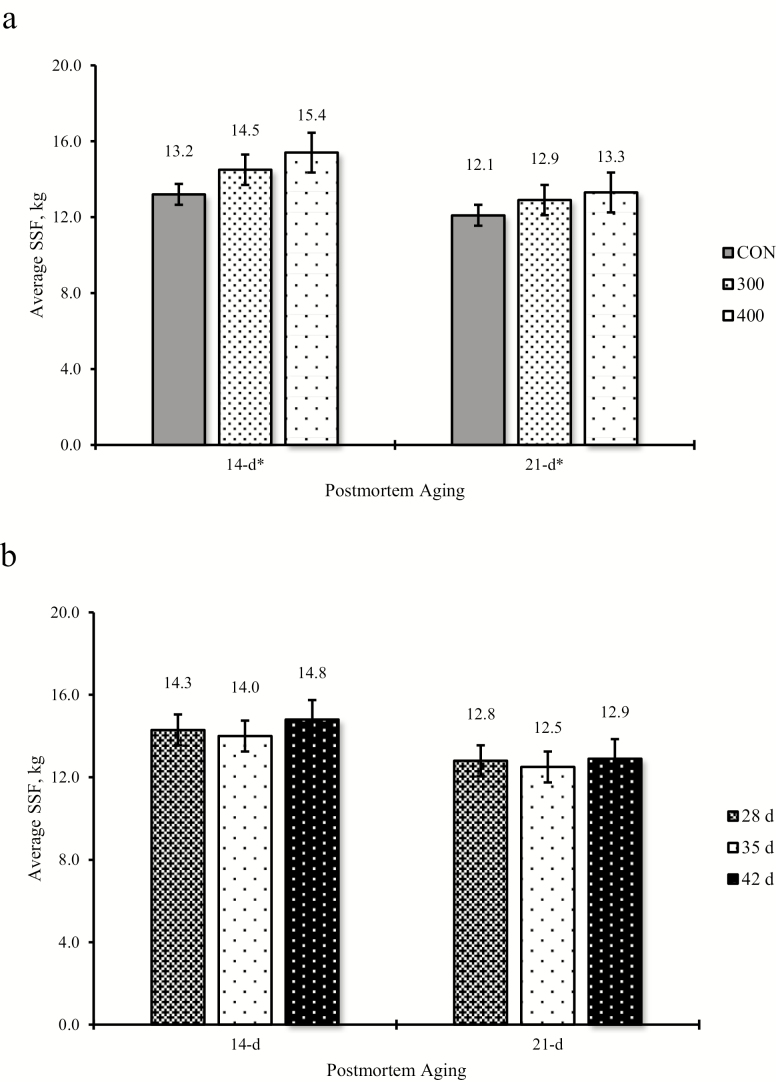
Effect of ractopamine hydrochloride dose (a) and duration (b) on average slice shear force (SSF) after 14- or 21-d aging. *Linear effect when *P* < 0.05. Fourteen-day average SEM = 0.43, 21-d average SEM = 0.36. Slice shear force was collected on 3 to 4 randomly selected steers from each pen (*n* = 417). Ractopamine hydrochloride was fed at 3 doses (0 [CON], 300, or 400 mg hd^−1^ d^−1^) at 3 durations (28, 35, or 42 d). ASTM (2011) specifications; <15.3 kg (very tender); 15.4 to 19.99 (very tender to tender); >20.0 kg (tough; not tender).

Average SSF for 14-d aged steaks increased (linear effect, *P* ≤ 0.01) with increasing RH dose (Fig. 2a). For steaks aged for 21 d, there was an increase (linear effect, *P* ≤ 0.01) in average SSF with increasing RH dose (Fig. 2a). Duration of RH feeding did not have an effect on average SSF values for either 14- or 21-d aging (Fig. 2b).

### Behavior and Mobility

There was no dose × duration interaction (*P* = 0.22) for initial chute temperament score. However, there was a dose × duration interaction (*P* ≤ 0.01) for the final chute temperament score. For RH dosages 0, 300, and 400 mg, means were 1.64, 1.71, and 1.58 when fed for 28 d; 1.66, 1.57, and 1.39 when fed for 35 d; and 1.65, 1.44, and 1.88 when fed for 42 d, respectively. Steers fed 400 mg·steer^−1^·d^−1^ for the 42-d duration had the greatest chute temperament score of all the treatments, whereas steers fed 400 mg·steer^−1^·d^−1^ for the 35-d duration had the lowest chute temperament score of all the treatments. Steers were generally calm to handle, and chute temperament score averaged 1.60 on day 0 and 1.61 for the final across all treatments (Table 5). Day 0 mobility and behavior scores were used in the covariate analysis to account for differences at day 0. There were no dose × duration interactions (*P* ≥ 0.26) for chute exit score. Chute exit scores decreased with increasing RH dose at both day 0 and final day (linear effect, *P* ≤ 0.01 and *P* = 0.02, respectively). At the start of RH feeding, there was a quadratic association (*P* ≤ 0.01) between chute exit score and increasing RH duration. However, final chute exit score was not affected (*P* ≥ 0.14) by RH feeding duration.

**Table 5.** Effects of feeding ractopamine hydrochloride on temperament and mobility of Holstein steers

	Dose				*P*-value		Duration				*P*-value	
Item	0	300	400	SEM	Linear	Quadratic	28	35	42	SEM	Linear	Quadratic
Pens	13	13	13				13	13	13			
Steers	237	239	241				238	239	240			
Chute temperament^1^												
Day 0^2^	1.68	1.61	1.51	0.09	0.11	0.52	1.60	1.61	1.51	0.09	0.35	0.09
Final^3^	1.65	1.57	1.62	0.25	0.55	0.45	1.64	1.54	1.66	0.25	0.88	0.10
Chute exit^4^												
Day 0	1.79	1.58	1.50	0.12	<0.001	0.95	1.67	1.47	1.74	0.12	0.51	<0.001
Final^5^	1.39	1.26	1.21	0.20	0.02	0.55	1.25	1.25	1.35	0.20	0.14	0.45
Pens	8	8	8				8	8	8			
Steers	137	135	138				137	136	136			
Stride length^6^, cm												
Day 0	40.8	41.4	41.7	0.03	0.37	0.76	43.3	40.4	40.6	0.30	0.02	0.31
Final	43.1	44.9	47.4	0.02	<0.001	0.96	43.2	47.9	44.9	0.20	0.37	0.02
Mobility^7^												
Day 0	1.17	1.13	1.14	0.06	0.45	0.67	1.16	1.13	1.15	0.06	0.79	0.66
Final	1.18	1.17	1.16	0.04	0.67	0.93	1.14	1.24	1.13	0.04	0.88	0.04
Harvest mobility^8^	1.32	1.39	1.37	0.06	0.19	0.45	1.32	1.41	1.35	0.06	0.53	0.11

^1^Steers were observed in a squeeze chute for 15 s after their head was caught, by one blinded, trained observer. The 1 to 4 point scoring system was adapted from Grandin (1995): 1 = calm, no movement; 2 = restless shifting; 3 = head throwing, squirming and occasionally shaking the squeeze chute; 4 = violently and continually shaking the squeeze chute.

^2^Respective duration ractopamine hydrochloride feeding day 0.

^3^Last day of ractopamine hydrochloride feeding.

^4^Steers were observed as steers left the squeeze chute by one trained individual. The 1 to 4 point scoring system was adapted from Grandin (1995): 1 = normal walk; 2 = trot or fast walk; 3 = run or sprint; 4 = leap or jump.

^5^Last day of ractopamine hydrochloride feeding. d 0 chute exit score was used as a covariate in the model (*P* = 0.01).

^6^Steers were observed for individual stride length while moving approximately 4 m from the squeeze chute by measuring the distance between the furthest back rear foot to the back of the forward rear foot when both hooves were in contact with the dirt surface.

^7^Steers were evaluated for individual mobility while moving approximately 30 ft from exiting the squeeze chute. The 1 to 4 point scoring system was adapted from Lily Edwards-Callaway, JBS: 1 = normal, fluid, even rhythm, and weight bearing on all 4 feet; 2 = slightly hesitant and stiff, shuffles feet, but still moves with the herd; 3 = obviously still and sore footed, reluctant to move, cannot keep up with the herd; 4 = extremely reluctant to move, animal refused to move when encouraged by a handler; any steps are short and very unsteady.

^8^Harvest mobility scores were observed at packing plant as steers were moved into harvest facility.

**Figure 3.**
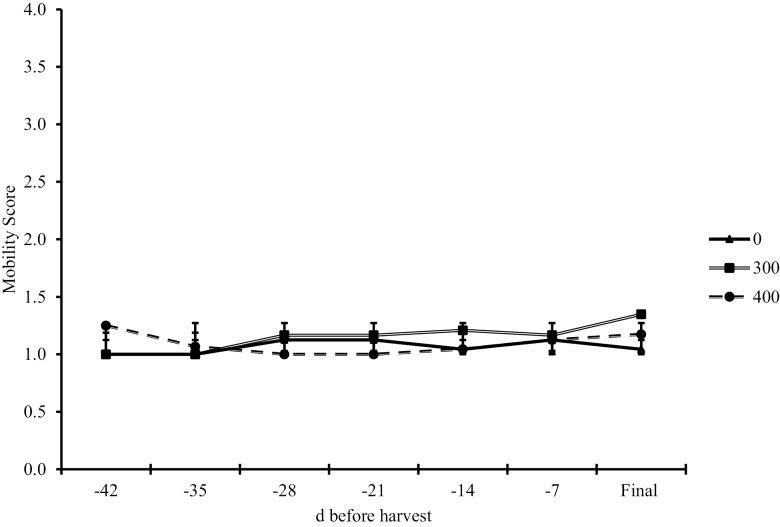
Effects of ractopamine hydrochloride fed at 0, 300, or 400 mg steer^−1^ d^−1^ on pen mobility scores when fed to Holstein steers. Ractopamine hydrochloride was fed at 3 doses (0, 300, or 400 mg hd^−1^ d^−1^) at 3 durations (28, 35, or 42 d). Steers were evaluated as a pen for mobility while moving from their home pens to the processing facility. The 1- to 4-point scoring system was adapted from Lily Edwards-Callaway; JBS. 1 = normal, fluid, even rhythm, and weight bearing on all 4 feet, 2 = slightly hesitant and stiff, shuffles feet, but still moves with the herd, 3 = obviously still and sore footed, reluctant to move, cannot keep up with the herd, 4 = extremely reluctant to move, animal refused to move when encouraged by a handler; any steps are short and very unsteady. SEM = 0.08. Dose, *P* = 0.18; Week, *P* = 0.16; Dose × Week, *P* = 0.11. Dose: linear, *P* = 0.26; quadratic, *P* = 0.14.

Final stride length was increased (linear effect, *P* ≤ 0.01) with increasing RH dose and responded quadratically (*P* = 0.02) with duration of feeding. Day 0 mobility score and harvest mobility were not affected by RH (*P* ≥ 0.45). Mobility score prior to shipping steers was greater (quadratic effect, *P* = 0.04) for the 35-d duration compared with the 28- and 42-d duration steers. Dose did not affect pen mobility scores throughout the RH feeding period (*P* ≥ 0.11; Fig. 2). For all steers, average mobility scores for all treatments was 1.17, indicating that steers moved fairly normal regardless of RH dose or treatment (Fig. 3).

## DISCUSSION

During May 2015, the feedlot received 12.5 cm more rainfall than the previous 10-yr average for that month. Due to deteriorating pen conditions, steers from Group 1, prior to their RH treatment period, were moved to larger holding pens while attempting to improve pen conditions. Steers were housed in larger holding pens by RH dose within block (21 steers per pen) so that 3 experimental pens (regardless of duration) were in holding pen for blocks 4 through 7. A minimum of 14 d prior to the start of the 42-d RH duration, steers were sorted and returned to the experimental pens where they were originally allocated (7 steers per pen). The 3 blocks initially started on RH remained in their original treatment pens for the duration of the experiment. Mader (2014) suggested that buildup of mud within a pen decreases performance, DMI, and welfare during any season. It is difficult to determine what impact, if any, the increased mud had on performance and carcass characteristics of steers in the present experiment.

In the present experiment, increasing dose of RH resulted in linear increases in live and carcass adjusted BW gain, ADG, and G:F. In addition, carcass weight gain, ADG, and G:F demonstrated a linear response when increasing dosage of RH. There was a 0.9 and 1.9 kg improvement in live and carcass-adjusted gain when 400 vs. 300 mg·steer^−1^·d^−1^ of RH was fed, respectively. Considering the linear increase in gain and efficiency, current market and other economic parameters should be evaluated to determine the most profitable dose. In spite of the muddy conditions, animal performance data from the present experiment are consistent with results from previous experiments (Bass et al., 2009; Vogel et al., 2009; Brown et al., 2014) when RH was fed to Holstein steers during the final 28 to 38 d of the feeding period. Previous studies have reported greater BW gain (4.3 to 8.0 kg), increased ADG (0.05 to 0.28 kg/d), and an improvement in G:F (14.2% to 16.6%) in calf-fed or yearling Holstein steers fed 200 to 300 mg·steer^−1^·d^−1^ of RH compared with steers not fed RH (Bass et al., 2009; Vogel et al., 2009; Brown et al., 2014). In the present experiment, BW was improved by 6.3 and 7.2 kg, ADG by 0.20 and 0.22 kg/d, and G:F was increased 20.8% and 21.7%, when steers were fed 300 and 400 mg·steer^−1^·d^−1^ of RH, respectively. The magnitude of response to RH on DMI in Holstein steers has been inconsistent. Vogel et al. (2009) reported a decrease in DMI in calf-fed Holsteins as dose of RH fed increased from 200 to 300 mg·steer^−1^·d^−1^. In the same experiment, DMI was increased in yearling Holstein steers fed 200 mg·steer^−1^·d^−1^ vs. the control. Factors contributing to variation in DMI across experiments are difficult to determine, but could include frequency of weighing, weather, adaptation to RH, and components of the diet, among others. Limited data are available comparing doses of RH in diets fed to Holstein steers. Vogel et al. (2009) fed calf-fed Holstein steers 200 vs. 300 mg·steer^−1^·d^−1^ of RH. The authors reported that increasing RH dose from 200 to 300 mg·steer^−1^·d^−1^ decreased DMI (0.41 kg/d), but did not affect ADG or G:F. In beef heifers, Quinn et al. (2008) reported an increase in carcass-adjusted gain and G:F when heifers were fed RH for 28 d compared with a control and increasing dose from 200 to 300 mg·heifer^−1^·d^−1^ decreased DMI, but did not affect feedlot performance.

The present experiment is the first to evaluate 0, 300 or 400 mg·steer^−1^·d^−1^ of RH fed for durations of 28, 35, or 42 d in Holstein steers. In beef steers, Abney et al. (2007) evaluated the effects of RH dose (0, 100 or 200 mg RH/steer daily) and duration (28, 35 or 42 d prior to harvest) on finishing performance and carcass characteristics. As duration of feeding RH increased, final BW, ADG, and G:F increased or tended to increase quadratically. These authors reported increases of 7.7 and 3.6 kg in final BW when steers were fed RH for 35 and 42 d, respectively, compared with feeding ractopamine for 28 d. In a recent experiment by Bittner et al. (2017), final and carcass-adjusted final BW were greater when 200 mg·steer^−1^·d^−1^ of RH was fed for 42 compared with 28 d. However, live and carcass-adjusted ADG was not different, and G:F was decreased when 200 mg·steer^−1^·d^−1^ of RH was fed for 42 compared with 28 d. In a second experiment by Bittner et al. (2017), dose × duration interactions were observed for final BW and G:F. At 28 d, steers fed 400 mg·steer^−1^·d^−1^ of RH had 6 kg greater final BW than steers fed 300 mg·steer^−1^·d^−1^. In contrast, when RH was fed for 42 d, steers fed 300 mg·steer^−1^·d^−1^ of RH had a 3 kg greater final BW than steers fed the 400 mg·steer^−1^·d^−1^ dose. Although not determined in the present experiment, the authors speculate that the difference in gain may be the results in sensitivity of the agonist receptor for RH. Increasing feeding duration of RH might decrease the efficiency of the product (NASEM, 2016).

Calculated carcass performance has been reported in the literature (Parr et al., 2011; Rathmann et al., 2012; Maxwell et al., 2014, 2015) for beef cattle. Estimating carcass-based performance is difficult because of the inability to measure carcass weight at the start of the feeding period, and as a result, initial HCW must be estimated. For the calculation of carcass-adjusted performance, the overall average dressing percentage of 60.41% was used. On a calculated carcass gain basis, steers fed 300 or 400 mg·steer^−1^·d^−1^ of RH had 0.12 and 0.18 kg/d greater carcass ADG than control steers. Comparing steers fed RH to control steers, the improvement in calculated carcass gain averaged 19.2% to 21.8% on a carcass-adjusted gain, and 18.6% on a live gain basis. Due to similarities in DMI, these magnitudes of difference also hold true for calculated carcass efficiency. Streeter et al. (2012) reported that the ratio of carcass gain to live gain was 88% for steers, regardless of technology use. Although the ratio was closer to 0.70 for Holstein steers in the present experiment, the efficiency in which live weight was transferred to carcass weight did not appear to change due to feeding RH, similar to the results of Streeter et al. (2012).

Carcass characteristics reported in the present experiment concur with those of Bass et al. (2009), Vogel et al. (2009), and Brown et al. (2014). They reported, increased HCW (3.1 to 8.2 kg), LM area (1.54 to 2.77 cm^2^), and decreased calculated YG (0.07 to 0.14 units) in carcasses from Holstein steers fed RH at a dosage of 200 to 300 mg·steer^−1^·d^−1^ compared with control steers. In the present experiment, HCW was increased 2.9 and 4.4 kg and LM area increased by 1.3 and 2.7 cm^2^ for steers fed 300 and 400 mg·steer^−1^·d^−1^ of RH, respectively. In addition, the proportion of carcasses in the USDA Yield Grade 2 category increased, and the number of carcasses in the USDA Yield Grade 3 category tended to decrease when dose of RH was increased. In the experiment by Vogel et al. (2009), increasing RH from 200 to 300 mg·steer^−1^·d^−1^ did not affect HCW or LM area in carcasses from calf-fed Holstein steers. Results from the present experiment suggest an increase in HCW and LM area as RH dose increases from 300 to 400 mg·steer^−1^·d^−1^ in Holstein steers. The effect of dose of RH on 12th-rib fat thickness and marbling scores have been inconsistent. In calf-fed Holstein steers, 12th-rib fat thickness was similar between steers fed 0 and 200 mg·steer^−1^·d^−1^ RH, but was least when 300 mg·steer^−1^·d^−1^ was fed (Vogel et al., 2009). Fat thickness and marbling scores were not affected by feeding RH in the studies by Bass et al. (2009) or Brown et al. (2014). In the present experiment, marbling score decreased linearly as RH dose increased; however, the distribution of USDA Quality Grades was not affected by RH dose. Duration of RH feeding had no effect on HCW, LM area, marbling score, 12th-rib fat thickness, or the distribution of USDA Yield Grades in the present experiment. Abney et al. (2007) reported HCW being 6 and 3 kg heavier for steers fed for 35 and 42 d, respectively, compared with steers fed for 28 d. Bittner et al. (2017) reported that feeding 400 mg·steer^−1^·d^−1^ of RH for 28 or 42 d resulted in increases of 7.6 and 8.9 kg, respectively, in HCW compared with steers fed 0 mg RH. In the present experiment, HCW over controls was linearly affected by dose, but was similar when RH was fed for 28, 35, or 42 d.

Martin et al. (2014) reported that steaks from calf-fed Holstein steers fed 300 mg·steer^−1^·d^−1^ of RH had greater SSF values than steaks from steers not fed RH. In addition, steaks from steers fed RH had greater SSF values regardless of postmortem aging length. Howard et al. (2014) evaluated the effects of feeding 0, 300, or 400 mg·steer^−1^·d^−1^ of RH to calf-fed Holstein steers the final 31 d of finishing. Steers fed RH produced steaks with SSF values greater than controls; however, no difference was detected between the two levels of RH at either 14- or 21-d aging. In the present experiment, compared to controls, the probability of steaks aged 14 d meeting the SSF requirements to be certified tender (SSF < 20 kg) was 0.85 and 0.83 in steers fed 300 or 400 mg·steer^−1^·d^−1^ of RH, respectively and after 21-d 0.97 and 0.92 in steers fed 300 or 400 mg·steer^−1^·d^−1^ of RH, respectively were certified tender. Increasing dose of RH, regardless of aging, increased average SSF values, but 88.6% and 98.0% of steaks had an SSF < 20.0 kg after 14- and 21-d aging, respectively. In addition, SSF value for steaks aged for 21 vs. 14 d was numerically less, especially for steaks from steers fed 400 mg·steer^−1^·d^−1^ of RH. Within the beef industry, the aging of beef carcasses is dependent on the cut of meat and facility preference. Savell et al. (2016) reported for ribeye steaks the average aging time was 29 d and the percentage of steaks that were aged less than 14 d was approximate 8.4%. Based on results from the present experiment and facility aging time of steaks, steaks become more tender as aging time increases, regardless of RH dosage prior to harvest. Results from the present experiment suggest that the percentage of steaks with ≥20.0 kg of SSF will increase with increasing dose of RH and some of the negative effect of increasing RH dose can be mitigated if steaks are aged for 21 d postmortem.

With the increased awareness of animal welfare, it is prudent to observe if βAA affect animal well-being. Lyles and Calvo-Lorenzo (2014) indicated there is little evidence of welfare implications of feeding βAA. Although a dose × duration interaction was observed, the present experiment did not detect a difference in chute temperament score related to dose of RH. Results from the present experiment are similar to Baszczak et al. (2006) who reported no changes in chute temperament score between steers supplemented with or without RH. In the present experiment, there were significant RH dose and duration effects on chute exit score on d 0 at the start of RH feeding. Therefore, day 0 chute exit score was included in the model as a covariate for final chute exit score. Chute exit score at the end of the RH feeding period decreased as RH dose increased in the present experiment. In the experiment by Baszczak et al. (2006), chute exit score was unaltered by βAA supplementation. Using the same chute temperament scoring as the present experiment, Hagenmaier et al. (2017) observed that prior to transport to the harvest facility, a greater percentage of beef steers not treated with RH had chute temperament scores and chute exit scores > 1 when compared with RH treated steers. Results from the present experiment suggest that dose and duration of RH feeding have little to no effect on chute temperament and exit scores in Holstein steers.

There are limited data on stride length in feedlot cattle receiving various growth-promoting technologies, although stride length, gait, and mobility are utilized within the dairy industry to identify lameness issues. When evaluating an animal’s gait, general stride length is shortened as mobility score increases due to the inability of the animal to move due to injury or illness. In healthy cattle, longer stride lengths are common among least mobility scores and indicate no injury or illness inhibiting normal movement. In Holstein dairy cows, longer stride lengths (Flowers and Weary, 2006) and decreased lameness issues (Telezhenko et al., 2017) were reported in cows moved on soil or rubber-based areas compared with concrete surfaces. In the present experiment, the alleyway where stride length was measured was dirt surfaced along with the majority of the experimental pen. It is unclear why increasing RH dose (linear effect) and increasing duration (quadratic effect) increased stride length in Holstein steers. Although data are limited in beef steers, Bernhard (2014) concluded that feeding zilpaterol hydrochloride did not affect step length. Based on results from the current experiment with Holstein steers and Bernhard (2014) with beef steers, regardless of cattle structure, the addition of growth-promoting additives (i.e., RH) to the diet does not affect stride length of the animal.

In the present experiment, mobility scores were observed as individual animals exited the squeeze chute. Dose of RH did not affect individual mobility scores captured at the end of the feeding period, although there was a quadratic increase in mobility score associated with duration of RH feeding. Using the same mobility scoring in a group scoring system, Hagenmaier et al. (2017) reported no difference in percentage of cattle with a mobility score > 1 when comparing RH treated and control cattle. In the experiment by Hagenmaier et al. (2017), beef steers were processed with either high-stress or low-stress handling methods, which may have affected the behavior and mobility results reported in their experiment. In the present experiment, steers were scored as each group was moved from their holding pens to the processing area, and were individually assigned a mobility score by the same technician as they were moved into the abattoir. Treatment had no effect on pen mobility score or mobility at harvest. Boyd et al. (2015) reported that although no impact was observed for feeding zilpaterol hydrochloride on cattle mobility scores, mobility scores decreased for all cattle at harvest.

The addition of RH the last 28 to 42 d on feed-in Holstein steer diets increased BW gain, ADG, G:F, HCW, and LM area. Results suggest feeding up to 400 mg·steer^−1^·d^−1^ will improve Holstein steer performance and carcass characteristics when RH. It should be noted the percentage of steaks classified as not tender or tough was 10 percentage points greater for steers fed 400 vs. 300 mg·steer^−1^·d^−1^ of RH after aging for 14 d but decreased to only 1.0 percentage point difference after 21 d of aging. We conclude feeding RH has little to minimal effects on animal behavior or mobility. Increasing dose of RH up to 400 mg·steer^−1^·d^−1^ fed for the last 28 to 42 d of the feeding period can improve live weight gain, efficiency, and carcass gain and leanness.
